# The effect of functional hearing loss and age on long- and short-term visuospatial memory: evidence from the UK biobank resource

**DOI:** 10.3389/fnagi.2014.00326

**Published:** 2014-12-09

**Authors:** Jerker Rönnberg, Staffan Hygge, Gitte Keidser, Mary Rudner

**Affiliations:** ^1^Linnaeus Centre HEAD, Department of Behavioural Sciences and Learning, Swedish Institute for Disability Research, Linköping UniversityLinköping, Sweden; ^2^Environmental Psychology, Faculty of Engineering and Sustainable Development, University of GävleGävle, Sweden; ^3^National Acoustic LaboratoriesSydney, Australia

**Keywords:** visuospatial tasks, memory systems, functional hearing loss, age, hearing aids

## Abstract

The UK Biobank offers cross-sectional epidemiological data collected on >500,000 individuals in the UK between 40 and 70 years of age. Using the UK Biobank data, the aim of this study was to investigate the effects of functional hearing loss and hearing aid usage on visuospatial memory function. This selection of variables resulted in a sub-sample of 138,098 participants after discarding extreme values. A digit triplets functional hearing test was used to divide the participants into three groups: poor, insufficient and normal hearers. We found negative relationships between functional hearing loss and both visuospatial working memory (i.e., a card pair matching task) and visuospatial, episodic long-term memory (i.e., a prospective memory task), with the strongest association for episodic long-term memory. The use of hearing aids showed a small positive effect for working memory performance for the poor hearers, but did not have any influence on episodic long-term memory. Age also showed strong main effects for both memory tasks and interacted with gender and education for the long-term memory task. Broader theoretical implications based on a memory systems approach will be discussed and compared to theoretical alternatives.

## Introduction

There is sufficient evidence to conclude that there is a connection between sensory decline and cognitive decline. Decline in one function is associated with decline in the other and the strength of the association has been empirically shown to increase with increasing age (Lindenberger and Baltes, 1994; Baltes and Lindenberger, 1997; Valentijn et al., 2005). This may suggest that there is some kind of common cause (e.g., neural degeneration) that explains the association, but more recent longitudinal evidence does not unequivocally support this hypothesis (Lindenberger and Ghisletta, 2009). Another explanation is that the sensory loss actually causes the cognitive decline (called the sensory deprivation hypothesis), and a third alternative is that cognitive decline drives sensory loss (Baltes and Lindenberger, 1997).

In this paper we focus on what might be dubbed the interactive hypothesis. Under this hypothesis, research has targeted mechanisms that underlie the online interaction (e.g., during speech understanding) between different hearing-related perceptual aspects on the one hand and cognitive aspects on the other. One such mechanism is perceptual stress or perceptual degradation, where it is typically assumed that even when stimuli are audible, the hearing loss affects the quality of encoding of memory items (e.g., Pichora-Fuller, 2003; McCoy et al., 2005). Another mechanism relates to the attentional costs that may be involved, implying that even a mild hearing loss draws on central attention resources, hence affecting memory encoding negatively (e.g., Sarampalis et al., 2009; Tun et al., 2009; Heinrich and Schneider, 2011). Still another possibility is that the long-term cognitive consequences of hearing loss strike selectively at different memory systems, even when audibility is high at testing (Rönnberg et al., 2011; Ng et al., 2013), and even when the to-be-remembered items are encoded in modalities other than audition (e.g., motor encoding, Rönnberg et al., 2011).

In this study, we pursue this memory systems approach with strictly non-auditory encoding conditions so as to minimize hearing-related perceptual encoding problems, hence making a conservative test of the set of hypotheses that hearing loss affects encoding more generally (i.e., independently of encoding conditions), that the locus of the effect is at the level of memory systems, and that there is selectivity in terms of which system is most affected. We outline the reasons for the predictions below:

In Rönnberg et al. (2011) it was found that hearing loss had a negative effect on both episodic and semantic long-term memory, but not on short-term/working memory. This held true even when chronological age was statistically controlled for and for tasks that did not rely solely on auditory encoding, thus minimizing the reliance on potential perceptual degradation (e.g., Schneider et al., 2002) or attentional effort (e.g., Tun et al., 2009). Using linear Structural Equation Models (SEM), Rönnberg et al. (2011) demonstrated that models that *combined* the degree of hearing loss with the degree of visual acuity did not make satisfactory predictions of memory decline for *any* memory system. Thus, the results suggest that relative decline in a memory system is tightly connected specifically to hearing loss rather than to sensory decline in general.

Rönnberg et al. (2011) explained their findings on the basis of relative use/disuse of memory systems, essentially stating that working or short-term memory is often occupied with storage of heard words and with reconstruction and repair of misheard words or sentences, whereas episodic long-term memory will become relatively less used in individuals with hearing loss because of the higher probability of mismatches (or no-matches) between input phonology and stored phonological representations of words in semantic long-term memory. Therefore, unlocking of the lexicon, and hence, episodic memory encoding/retrieval, will occur to a lesser extent for individuals with hearing loss than for individuals with normal hearing while working or short-term memory will be engaged to the same extent, if not more.

The prediction regarding semantic long-term memory based on a use/disuse concept is less clear because it could be argued that semantic and contextual knowledge would have to be used more than episodic memory to compensate for misheard or non-matching words (Rönnberg et al., 2008, 2011, 2013). This is evident e.g., in studies of false hearing, where older adults rely to a larger extent on context (Rogers et al., 2012). However, the data suggest a decline due to hearing loss even for semantic memory, especially for phonologically sensitive fluency tasks (Rönnberg et al., 2011) and for nonword recall tasks (Janse and Newman, 2013).

Testing the short-term/working memory system in more detail, Verhaegen et al. (2014) have recently shown that, especially in auditory short-term memory tasks that rely on serial recall of words, there is an effect of hearing loss that is not related to age (see also Pichora-Fuller et al., 1995; van Boxtel et al., 2000; Schneider et al., 2010). This effect occurs even when the hearing loss of the study sample was mild (25–30 dB). They also argued that the results did not support the neural degeneration hypothesis (i.e., an example of a common cause) since young and old participants with hearing loss performed on a par, thus leaving most of the explanatory power to hearing status and not to age, as both groups were outperformed by a third group of young individuals with normal hearing. It was further reasoned that because speeded non-word repetition was intact even in the hearing-impaired groups, the actual perceptual processes were intact. It was proposed, in line with several other studies (cf. McCoy et al., 2005; Wingfield et al., 2005; Tun et al., 2009; Piquado et al., 2010), that increased demands on attention may instead be a plausible hypothesis regarding the mechanism involved (Verhaegen et al., 2014).

In the current study, based on a large sample (*N* = 138,098) of people not using hearing aids from the much larger UK Biobank Resource (*N* > 500,000), we therefore focused on the effects of hearing loss and age on memory tasks that were *not* confounded by possible auditory perceptual degradation, or by attentional demands related to hearing difficulties, strictly testing the memory systems hypothesis.

Testing the memory systems hypothesis, we used two types of memory tasks, tapping visuo-spatial working memory and visuo-spatial episodic long-term memory, respectively. The working memory task was a card-pair matching game in which participants had to remember cards that were the same (pictures of ordinary animals/objects like e.g., cat/ball) after having had a short inspection time. Two versions of the task were employed, an easy one with three pairs (which was considered to be a warm-up task), and a more difficult one with six pairs (loading highly on visuospatial memory). Thus, we opted for the six pair version in our analysis to maximise the demands on working memory.

As a proxy for episodic long-term memory function and to determine whether we could replicate the negative effect of hearing loss on episodic long-term memory (Rönnberg et al., 2011), we used a prospective long-term memory task, a task that has a clear episodic long-term memory component (Burgess and Shallice, 1997). At the beginning of the session, subjects were given instructions (written on the computer screen) stating that they were to touch a colored shape when prompted at the end of the session. Crucially, they were also informed that the prompt on the screen would say *blue square*, but as a prospective memory test, they should instead touch the *orange circle*.

Although short-term memory has been shown to be affected by hearing loss (Verhaegen et al., 2014), it should be noted that the data by Rönnberg et al. (2011) suggest that working memory/short-term memory is *relatively* less affected by hearing loss than episodic long-term memory. This is the central hypothesis in the present study. Thus, by using the two visuospatial memory indices briefly described above, we were able to make a very conservative test of the hypothesis that functional hearing loss is more strongly related to episodic long-term memory decline than to short-term or working memory decline and that these declines are not caused by perceptual degradation or lack of attention resources. Semantic memory measures were not included in the present study.

In a separate sample from the UK Biobank resource (*N* = 3751, see “Additional Analyses” section below), we also checked for the effects of hearing aid usage, with the hypothesis that this may have a protective effect against memory decline (Rönnberg et al., 2011). This has not been examined in detail in previous studies: for example, in Rönnberg et al. (2011) we only used data from individuals with hearing loss who were also users of hearing aids, in the seminal studies reported by Baltes and Lindenberger (1997) hearing aid usage was not separately accounted for (see Arlinger, 2003), and in the Verhaegen et al. (2014) study, the participant sample did *not* use hearing aids.

Finally, as we used visuospatial memory tests, we also deemed it appropriate to use two simple measures of visual acuity/vision problems as another sensory-specific possibility to explain any hearing loss-related decline. In this way we can cast more light on the influential Baltes-Lindenberger common-cause hypothesis.

The sample from the UK Biobank resource used in the present study is extremely large compared to that used in any other study in the literature on this topic. It will guarantee statistical power and generalizability.

## Methods

### Overall sample

The UK Biobank resource consists of data obtained from more than 500,000 participants. In the present study, we excluded participants who were born outside of the UK and the Republic of Ireland, as unknown language and cultural differences may significantly affect their cognitive abilities. We also excluded participants whose data sets were incomplete across measures of hearing and cognition. In addition, in the first main analyses we did not include hearing aid users (HAUse). This resulted in a study sample of 138,098 participants. Among these, 75,065 were females and 63,033 were male; giving a slightly skewed ratio of 54/46 (%). Age ranged from 39–70 years reflecting the UK Biobank population as a whole.

### Subjective reports

The UK Biobank population also answered yes/no questions about “difficulty with hearing in general” (*N* = 439,510) and “difficulty following a conversation if there is background noise (such as TV, radio, children playing)” (*N* = 448,416). Among the UK population, 114,717 (25%) reported having general difficulty with hearing, 169,055 (37%) had difficulty hearing in noise, and 14,010 (3%) wore hearing aids. In our sample of 138,098 persons, we had data for 130,206 on reported general difficulty with hearing, and 24% reported such difficulty. For hearing in noise, we had data for 134,673 persons and 34% reported difficulties with that. With respect to hearing aid usage, 3751 persons (2.6%) in our sample reported wearing a hearing aid.

Furthermore, participants were asked whether they wore glasses (no/yes) and whether they had diagnosed eye problems/disorders other than those corrected for by the use of glasses. In our sub-sample of 138,098, 89% (of 137,978) reported having eye-glasses and 88% (of 101,845) reported having no additional eye-problems.

Participants were also asked which of six qualifications they had obtained. To simplify further analyses, a new highest level of qualification variable was created that assumes that a College or University degree (rated 1) > A levels/AS levels (rated 2) > O levels/GSEs (rated 3) > CSEs (rated 4) > NVQ or HND or HNC (rated 5) > Other professional qualifications; e.g., nursing or teaching (rated 6). In our sub-sample, we had valid values for 116,947 on qualification and the distribution across qualification levels 1–6 was 38.8%, 13.8%, 26.6%, 7.1%, 7.7%, and 6.0%, respectively.

The study presented here is covered by a Research Tissue Bank approval obtained by UK Biobank from its governing Research Ethics Committee, as recommended by the National Research Ethics Service.

### Tests

Participants attended 1 of 22 assessment centers spread throughout the UK. All test data used in this study were obtained through a self-administered program running of a computer with a touch screen that collected responses to questionnaires and tests on hearing in noise and cognition. Incomplete data sets were collected as it was possible for participants to be selective in which questionnaires and tests they responded to.

#### The digit triplets test (DTT)

The participants completed a functional hearing test in which they were presented with digit triplets in a steady state, speech-shaped noise (Smits et al., 2004) and had to enter (on a numberpad shown on the touchscreen) which three digits they had heard (forced choice). The speech reception threshold in noise (SRTn) was the SNR arrived at after 15 presentations, during which noise was adaptively changed after each presentation depending on whether the three digits were correctly identified or not. These SNR could vary between −12 and +8 dB, where a high and positive score indicated worse hearing. Each ear was tested separately (unaided) under headphones. As a first step a best ear SRTn variable was created to be used in further analyses. One reason for choosing the best ear is that it dominates auditory function in daily life, and is typically used in insurance compensation for assessment of e.g., occupational hearing loss (Dobie, 1996, see also Dawes et al. (2014)). For those who only completed the test on one ear, it is assumed that this was the better ear, and this result is recorded. As a second step, we classified the participants on the basis of the criteria used by Dawes et al. (2014), where “normal” hearing was assumed for SRTn values below −5.5 dB, “insufficient” hearing as −5.5 to −3.5 dB, and “poor” hearing as a threshold above −3.5 dB (variable was denoted Hear). This classification, in turn, was based on earlier work within the HearCom project (Smits et al., 2004; Vlaming et al., 2011).

Smits et al. (2004) found a relatively high correlation between the Dutch DTT and pure tone audiometry of *r* = 0.77. One reason for a lack of perfect correlation is that people with similar audiograms can have different psychoacoustic profiles (e.g., individual differences in frequency and temporal resolution) and hence perform differently when listening to speech in noise. Therefore, it seems reasonable that DTT also has been found to correlate highly with speech-in noise-recognition measures (such as with Plomp and Mimpen (1979) Sentences in Noise; *r* = 0.85; Smits et al., 2004). Together, the DTT can be considered as a functional hearing test (Dawes et al., 2014); see also the General Discussion section below.

#### Cognitive tests

Four tests of cognitive function were performed in the following order: (1) Prospective Memory test: Shape—Part 1; (2) Pairs memory test; (3) Verbal Reasoning test; (4) Reaction time: Snap; (5) Prospective Memory test: Shape—Part 2. We here describe the pairs matching and the prospective memory tests, as they are used for the short-term—long-term memory distinction relevant to this paper. Data on reverse digit span were also available from the UK Biobank resource but were not used in the present study with its focus on visuo-spatial memory function.

##### Pairs memory test: visuospatial working memory (VSWM)

VSWM was measured with a pairs matching game. Participants were presented first with a round of three pairs of cards depicting different designs of objects and then, twice, with a round of six pairs of cards. The layout was purely random each time. There were no specific selection criteria applied to choosing the designs of the pictures other than that they should look reasonably distinct. Thus, there were no systematic phonological or semantic relationships between the English lexical labels of the pairs of objects. During each round, the pictures were turned over after a short inspection period. The 2 × 3 layout was shown for 3 s before pointing and the 2 × 6 layout was shown for 5 s. The participants were asked to identify as many pairs as possible with the fewest attempts by touching “pairs” of the same object on the screen. When the participant made an error, this was indicated by the word “miss” appearing at the center of the screen. When the participant gave a correct answer, the word “pair” would appear on the screen. For each correctly identified pair, the cards were removed and two blank spaces were left in the position where they had previously been placed. The participants could continue until they had identified all pairs. Time allowed for matching of pairs was unrestricted. The participants were allowed to continue until they had discovered all pairs correctly. The dependent variable is thus the number of errors made before all the pairs had been matched. We considered the three-pairs round a warm-up trial for the six pairs round, which constituted the dependent variable.

##### Prospective long-term memory (PLTM)

PLTM consisted of two parts:

*Part 1*. The initial instruction to the participant was the following: “At the end of the games we will show you four colored shapes and ask you to touch the Blue Square. However, to test your memory, we want you to actually touch the Orange Circle instead. Once the “Next” button was touched, a hidden timer was started to record the delay interval until the answer to this question (asked after the reaction time test) was requested. Then the Pairs matching test, the Fluid intelligence test and the Reaction time (Snap) test were performed.

*Part 2*. After the Reaction time (Snap) test was finished, the following text was shown to the participant: *“That’s the last game. Just one more thing left to do…”*. The participant then selected “Next”; and the Shapes screen appeared with the text: *“Please touch the Blue Square then touch the “Next” button”* was presented. At this point the delay interval timing ended. If the participant touched any of the symbols it was highlighted by surrounding it in a yellow box. If the participant touched the Next button without having highlighted a symbol they were shown the message: *“Please touch a symbol (a colored shape) before touching the “Next” button”* If the participant then touched any symbol other than the Blue Square, then Next, the test ended. If the participant touched the Blue Square, they were prompted with the message: *“At the start of the games we asked you to remember to touch a different symbol when this screen appeared. Please try to remember which symbol it was and touch it now”*. If the participant touched the Blue Square again then this message was repeated (*ad infinitum*), otherwise the program accepted their new selection and the test ended. The dependent variable was scored in three steps: correct at first attempt, correct at a subsequent attempt, and not correct at first or following attempts (which were given the scores 1, 2, and 3, respectively).

### Rationale for the statistical analyses

For the memory measures, logarithmic transformation of the number of errors made in VSWM and the errors scores in PLTM were computed (for both measures: natural logarithm of *x* + 1) to counteract the skewed distribution of the raw scores. Also, for the analyses of the VSWM and PLTM tasks, individuals with values above the 99th percentile on the six pairs matching tasks were excluded to build in a safeguard against outliers. Our initial analyses were also restricted to participants who did not use hearing aids.

To be able to compare error rates on the dependent variables VSWM and PLTM in the ANOVAs, rather than in regression analyses with dummy coding of the interactions, the age and the hearing variables were divided into sub-groups. Our aim was to have at least about 100 observations for each combination of age and hearing status. With the functional hearing status variable already divided into three groups (Good, Insufficient, and Poor), as suggested by Dawes et al. (2014); see also Smits et al. (2004)), and outlined above in The Digit-Triplets Test (DTT) section, a choice had to be made about age-group spans.

We preferred four age spans, and that the two middle spans would be 10 years. With hearing status groups already defined, the pragmatic solution was to move the two middle 10 year age spans down from the maximum age of 70 years in our sample, and ensure that the *N* in the smallest Age × Hearing status groups were ≈ 100 or more. With these criteria our oldest group was defined as >67 years, and the youngest as <48 years, with two 10-year age spans in between.

## Results

The Age by Hearing status distribution is shown in Table 1 of our *N* = 138,098 in our subsample. Table 1 also shows the defining criteria for the three hearing status groups: Normal, Insufficient, and Poor.

**Table 1:** **Number of persons in Age-groups and the three-step functional hearing status groups**.

		Hear			
	Age	Normal < −5.5	Insuff −5.5 to −3.5	Poor > −3.5	Total
1	<48	23147	881	90	24118
2	48–57	38724	2369	197	41290
3	58–67	55617	7340	835	63792
4	>67	7113	1567	218	8898
Total		124601	12157	1340	138098

Table 2 shows the dichotomized fractions of men and people with an education other than University, College, A level, AS level in the Age × Hearing status groups. These fractions do not vary substantially between sub-groups, but the means in the groups were statistically evaluated in our subsequent analyses (see below under Section Additional Analyses).

**Table 2:** **Proportions of men (1st fraction in each cell of the table) and proportions of persons with an education other than University, College, A level, AS level (2nd fraction) in the Age by Hearing status groups**.

		Hear						
	Age	Normal < −5.5		Insuff −5.5 to −3.5		Poor > −3.5		Total	
		Men	LoEduc	Men	LoEduc	Men	LoEduc	Men	LoEduc
1	<48	0.45	0.44	0.42	0.54	0.37	0.66	0.45	0.45
2	48–57	0.43	0.45	0.40	0.50	0.46	0.55	0.43	0.45
3	58–67	0.47	0.49	0.47	0.54	0.54	0.61	0.47	0.59
4	>67	0.49	0.54	0.49	0.58	0.57	0.64	0.49	0.55
Total		0.46	0.47	0.46	0.53	0.52	0.61	0.46	0.47

*Note. Fractions (0.0–1.0) of men and persons with an education other than University, College, A level, AS level in the Age by Hearing groups. For the fraction of men there are valid observations for the same 138,098 persons as in our standard sub-sample, but for education the total number is 116,947*.

We also decided to take a parametric approach to how to treat the logarithmic error scores for VSWM and PLTM. The basic issue is whether it can be justified to treat the scores as being on an interval scale, and reanalyze them with parametric tests, such as ANOVA, or whether the data only meet ordinal scale properties and should thus be subjected to non-parametric tests. We concluded that an ANOVA approach is justified, but we will discuss the pros and cons of that at the end of the Results Section and also provide non-parametric analyses of our data to support the parametric statistical analyses.

### Effects of hearing loss and age on performance in the two memory tests

**Figure 1:**
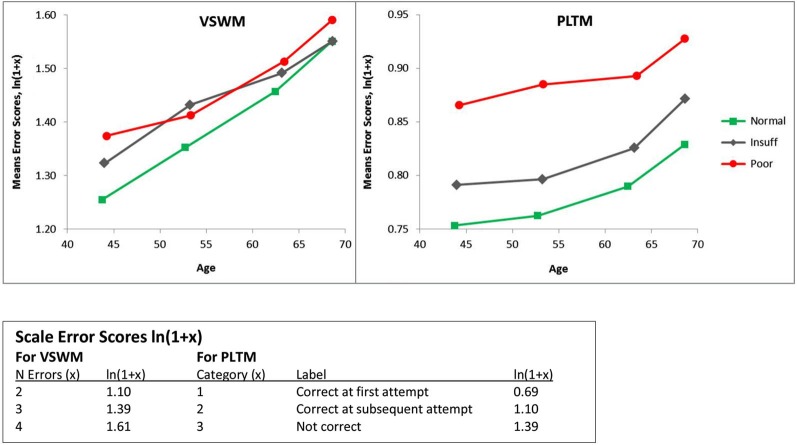
**Mean error scores (ln(1 + *x*)) plotted as a function of age and hearing according to Dawes et al. (2014), called Hear, with categories in SRT dB: Normal = < −5.5 Insuff = −5.5 to −3.5, Poor > −3.5)**. VSWM = The six-pairs picture matching task, PLTM = The prospective memory task. Note that as the *x*-axis is the actual mean ages in the age-groups, the slopes of the lines between the age-groups are on a comparable scale. This also explains why the *y*-values are not on the same vertical age-line.

Figure 1 presents the mean error scores (ln(1 + x)) plotted as a function of age and hearing according to Dawes et al. (2014), called Hear, with categories in SRT dB: Normal = <−5.5 Insuff = −5.5 to −3.5, Poor > −3.5). The left panel presents the data for VSWM and the right panel gives the data for PLTM. The ANOVAs were computed separately for VSWM and PLTM with Hear and Age as independent between-person factors. As can be seen from Figure 1 and as confirmed by the ANOVAs (see Table 3) there are significant effects of both Hear and Age. The Age effect is about equal in terms of *F*-values for the two memory tests, but the effect of Hear for PLTM appears to be stronger than it is for VSWM. Also, there is a significant interaction Hear × Age for VSWM, but not for PLTM.

**Table 3:** **F-tables for VSWM (upper panel) and PLTM (lower panel) by Hear and Age for the values given in Figure 1**.

	Sum of squares	*df*	Mean square	*F*	Sign. *p* =	Observed power
**VSWM source**
Hear	15.822	2	7.911	21.085	0.000	1.000
Age	41.860	3	13.953	37.189	0.000	1.000
Hear*Age	6.659	6	1.110	2.958	0.007	0.906
Error	51810.502	138086	0.375
**PLTM source**
Hear	16.861	2	8.431	243.940	0.000	1.000
Age	4.186	3	1.395	40.376	0.000	1.000
Hear*Age	0.146	6	0.024	0.705	0.645	0.285
Error	4772.280	138086	0.035

Thus, PLTM seems to be more sensitive to functional hearing status and judging from Figure 1, the dominating difference is between the poor and the insufficient hearers. To statistically corroborate this difference we conducted follow-up ANOVAs on the 12,157 insufficient hearers and compared them with the 1340 poor hearers. For PLTM, there was a marked difference between the poor and insufficient hearers, *F*_(1,13489)_ = 68.9, *p* < 0.000, between the normal and insufficient hearers, *F*_(1,136750)_ = 256.6, *p* < 0.000, and a significant effect of Age, *F*_(3,13489)_ = 12.18, *p* < 0.000, but no significant effect of their interaction (*F* < 1). For VSWM, there was no significant difference between the poor and insufficient hearers, (*F* < 1), a main effect of Age, *F*_(3,13489)_ = 12.81, *p* < 0.000, and no significant interaction (*F* < 1).

Thus, the ANOVAs and the pattern of simple main effects results strongly support the conclusion that there is a crucial difference in the pattern of age-related performance between PLTM and VSWM, especially when comparing the poor and insufficient hearers. Poor compared to insufficient hearing is markedly more deleterious to PLTM than it is to VSWM.

### Power and effect size

In Table 3 it can also be noted that the observed power is very high because of the large samples. Effect sizes (Cohen’s d) were calculated for pairwise comparisons between levels of Age and Hear for VSWM and PLTM, respectively, and are shown in Table 4.

**Table 4:** **Effect sizes (Cohen’s d) for VSWM and PLTM between adjacent levels and the highest vs. lowest levels of Age and Hear, for the same analyses shown in Table 3 and Figure 1**.

Age, years	Cohen’s d	
	VSWM	PLTM
<48 vs. 48–57	0.162	0.059
48–57 vs. 58–67	0.171	0.162
58–67 vs. >67	0.146	0.214
<48 vs. >67	0.478	0.461
**Hear**
Normal vs. Insufficient	0.134	0.250
Insufficient vs. Poor	0.041	0.324
Normal vs. Poor	0.175	0.646

*Note. The values in the Table can be compared to Cohen’s (1988) proposed rules of thumb for interpreting effect sizes: a “small” effect size is 0.20, a “medium” effect size is 0.50, and a “large” effect size is 0.80*.

As shown in Table 4, the effect sizes are mostly small (<0.20), but the effect of Hear is systematically greater and in the medium range for PLTM than VSWM. Particularly, the effect size of the comparison between normal and poor hearers for PLTM exceeds medium (>0.50), which is quite impressive with such a large sample. However, the effect sizes for the comparisons normal vs. insufficient hearers and insufficient and poor hearers were 0.25 and 0.32, respectively, which is closer to the small effect size.

Therefore, effects sizes are quite in line with the results from the separate ANOVAs, which showed large effects of both Hear and Age, the Age effect being about equal for the VSWM and PLTM, but also that the Hear effects were larger for PLTM than for VSWM.

### Additional analyses

To assess whether using a hearing aid modulated memory decline, we computed separate ANOVAs on the following sub-sample: for a total of 3751 of HAUse we had data on their Age and Hearing status, as well as on their scores within the 99th percentile on the memory tasks. Of these, 2139 were normal hearers (57%, out of 3751 HAUse), 1080 insufficient hearers (29%), and 532 were poor hearers (14%).

**Figure 2:**
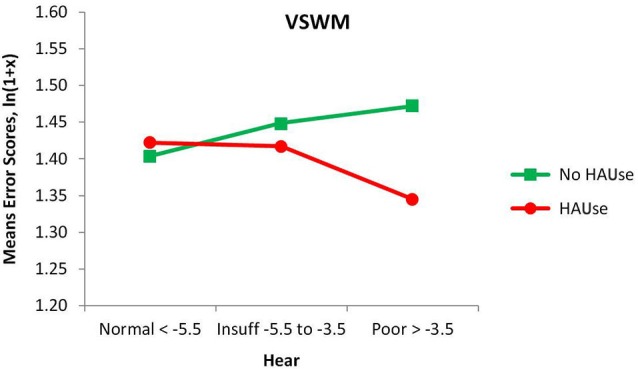
**Mean error scores (ln(1 + x)) for VSWM plotted as a function of hearing and the use of hearing aids**.

When adding HAUse as a separate third variable to Age and Hear in our separate ANOVAs, we noted a beneficial main effect of HAUse, shown as a reduction in the number of errors for VSWM for HAUse compared to non-users (*F*_(1,141825)_ = 4.86, *p* < 0.05). For VSWM there was also a significant interaction Hear × HAUse, *F*_(2,141825)_ = 4.20, *p* < 0.05, see Figure 2. A test of the simple main effects of HAUse indicated at significant difference between HA-users and No HA-user with poor hearing, *F*_(1,141825)_ = 7.10, *p* < 0.01 (with a Cohen’s d effect size of = 0.185) but not at the other two levels of hearing (*F* < 1). Thus, for VSWM the results indicated that for the normal hearers there was not much of a difference between those with and without hearing aids, but with increasing hearing loss the degree of “protection” against memory errors afforded by wearing hearing aids increased (see Figure 2). However, the effect size is relatively low, but inspecting the 95% confidence intervals for the means of the three levels of Hear in Figure 2 for the HA-users indicated that the mean for the poor hearers was outside the lower bounds of the means for the normal and insufficient hearers.

For PLTM, there was no main effect of HAUse, (*F* < 1), and no significant interaction Hear × HAUse (*F* < 1), but there was a significant interaction Age × HAUse, *F*_(3,141825)_ = 6.05, *p* < 0.000, which was specified by the interaction Hear × Age × HAUse, *F*_(6,141825)_ = 3.06, *p* < 0.01, (not given in any figure) showing that the poor hearers with hearing aids in the youngest group have markedly higher error scores than was the case for the other HAUse (their value of 0.967 is far above the upper 95% confidence limits for all of the other 11 Age × Hear groups with hearing aids. However, a warning is in place for this group, as it has the lowest *N* in that analysis, only 22). Thus, generally speaking, PLTM was not positively affected by the use of hearing aids, but for VSWM we could observe some more “protection” against making errors, as is suggested from the HAUse × Hear interaction in Figure 2. However, two points should be noted about this interaction: one is that we had so called normal hearers who used hearing aids. The fact that they seek treatment with presumably very mild or non-existent functional hearing loss is usually because of some other kind of communication difficulties. If the cochlear function does not contribute to these problems, we suggest that there are some underlying central processing or cognitive defects that contribute to the person’s experiences of having difficulties with communication. Second, we cannot be sure about causality (see more under Section General Discussion).

To eliminate Gender and Education (dichotomized as in Table 2) as confounders (cf. Table 2), we added these two independent variables to Age and Hear in a MANOVA, ending up with *N* = 116,947, as in Table 2. For VSWM there were no significant main effects or interactions involving Gender and/or Education. For PLTM there was a main effect of Education, *F*_(1,116899)_ = 85.19, *p* < 0.001, and an interaction Hear × Education, *F*_(2,116899)_ = 7.73, *p* < 0.001. These effects indicated that the persons with a lower education made more errors, and that this disadvantage was more marked for those with poor hearing. The 95% confidence interval for the poor group included the insufficient group for those with a higher education, but for those with a lower education, the insufficient group was lower by far in errors and outside the 95% confidence interval for the poor group. However, we cannot be conclusive about education *causing* better episodic long-term memory, but there are studies that suggest that schooling affects brain function and cognition many decades after schooling has terminated (Glymour et al., 2008; Nyberg et al., 2012).

For PLTM, there was also an interaction Age × Gender × Education, *F*_(3,116899)_ = 2.74, *p* < 0.05, meaning that males with lower education and in the age range 48–57 years, made more errors than women in the same group. However, caution should be observed when interpreting these results as the number of persons in 4 of the 48 (= 4 × 3 × 2 × 2) cells come as low as *n* < 30, particularly for the youngest and oldest poor hearers with high education.

Furthermore, replacing Hearing status in the original ANOVAs with the binary scored subjective reports of hearing difficulty and hearing difficulty in noise, did not yield any significant main effects or interaction (all *F*s < 1.97).

We also tested whether using eyeglasses or having reported eye problems had any association with the memory data but found no such relationships. Thus, it is mainly the objectively measured functional hearing loss (the SRTn for the DTT) that accounts for the observed memory declines.

### Probing the categorization of hearing status

To safeguard against missing some more delicate and detailed effects when a rather crude hearing criterion like the three-step Hear-distinction was employed, an analysis with a four-step hearing criterion (Hear4) was also performed. In this four-step criterion the extreme groups were the same as in the original Hear4 criterion, but the former middle-group (Insuff) was split into two groups, Insuff1 (SRT −5.5 to −5.0) and Insuff2 (SRT > −5.0 to 3.5). The number of persons are shown in Table 5.

**Table 5:** **Number of persons in Age-groups and the four-step Hearing status groups**.

		Hear4				
	Age	Normal < −5.5	Insuff1 −5.5–−3.5	Insuff2 −5.5–−3.5	Poor > −3.5	Total
1	< 48	23147	447	434	90	24118
2	48–57	38724	1119	1250	197	41290
3	58–67	55617	3175	4165	835	63792
4	> 67	7113	574	993	218	8898
Total		124601	5315	6842	1340	138098

**Figure 3:**
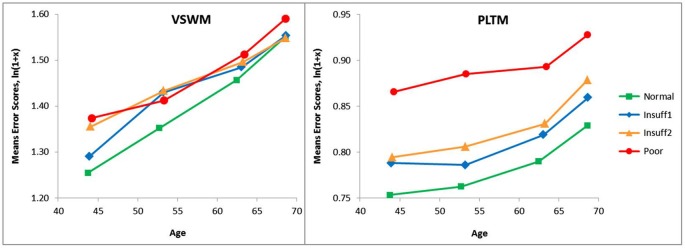
**Same as Figure 1 except that a four-step (Hear4) hearing grouping was employed. Categories in SRT dB: Normal = < −5.5 Insuff1 = −5.5 to −5.0, Insuff2 = −5.0 to −3.5 Poor > −3.5**. The Poor and Normal hearing group are the same as in Figure 1, and their lines have the same legends.

The results of the Hear4 grouping is depicted in Figure 3, which has the same y-axis as Figure 1, to make a visual inspection easy. However, the Hear4 grouping did not change the pattern of significant effects in the overall ANOVA already reported above in Table 3.

As can be seen when comparing Figure 1 and Figure 3, the splitting of the Hear insufficient group into two groups, did not reveal that Insuff2 approached the group with the poorest hearers. Insuff2 remained close to Insuff1 in performance on the two memory measures. This indicates that the pronounced problems with memory are mainly restricted to the 1% of the sample that has the worst hearing.

**Figure 4:**
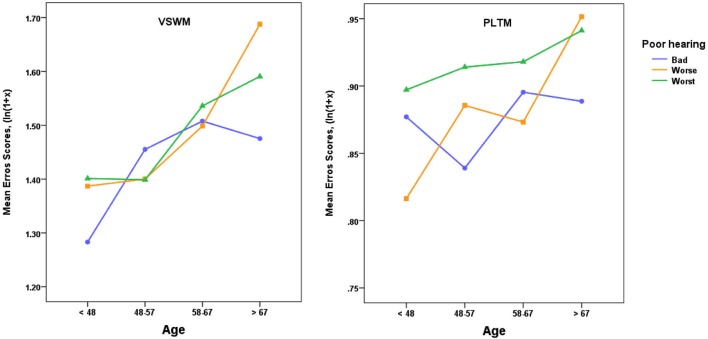
**Mean error scores (ln(1 + *x*)) plotted as a function of age and three levels of poor hearing, with categories in SRT dB: Bad = > −3.5 to ≤ −3.0, Worse = > −3.0 to ≤ −1.0, Worst = > −1.0**. VSWM = The six-pairs picture matching task, PLTM = The prospective memory task. For VSWM there was only a significant effect of Age (see text) and for PLTM there was a significant difference between the Bad and Worst groups, one-tailed *t*_(789)_ = 1.78, *p* < 0.05.

In a similar vein, we also probed what would happen to the scores for VSWM and PLTM when the group with poor hearers (*N* = 1 340) was divided into three poor hearing groups (Bad, Worse, Worst, see Figure 4 for hearing criteria, *N*s = 369, 549, 422 respectively). The results are shown in Figure 4, and the corresponding ANOVAs indicated that the only significant effect for VSWM was as a main effect of Age, *F*_(2,1328)_ = 3.25, *p* < 0.05. For PLTM there was no significant effect of Age (*p* > 0.10), but as indicated in Figure 4, the average errors in the worst sub-group of the poor hearers were higher than in the bad group. This difference was significant in a one-tailed *t*-test, *t*_(789)_ = 1.78, *p* < 0.05, but Cohen’s d was low (0.127).

Thus, a more fine-grained sub-grouping of the poor hearers pinpoints the extremely poor hearers, the worst group, as responsible for a significant share of the increase in error scores for PLTM, but not to the same extent for VSWM.

The general result of these analyses is that the effects of functional hearing loss are robust and prominent for episodic long-term memory, and driven by extremely poor hearers. Being a hearing aid user had no effect on the association between hearing and episodic long-term memory, but did influence the association between hearing and working memory; hearing aid users among poor hearers performed better than non-users. Education and gender modulated the episodic long-term memory decline but not working memory. Age affected both memory systems negatively, but interacted with gender and education only for episodic long-term memory.

### Parametric and non-parametric testing of VSWM and PLTM

There may be some doubt as to whether the scale properties of our measures of VSWM and PLTM meet the assumptions for a parametric ANOVA-test.

However, ANOVAs are known to be robust against violations of the underlying assumptions (discussed in several elementary text books in statistics, e.g., Howell, 2007). A normal distribution is not necessary, and testing skewed distribution against each other may be acceptable if the distributions have the same kind of skewness. Histograms of our VSWM scores showed a unimodal symmetric distribution. The PLTM measure showed a skewed distribution with more observation at the lower end of the scale. The VSWM measure showed a unimodal symmetric distribution, if the interval band width was set to 0.5.

We also made analyses of VSWM and PLTM with the SPSS Generalized Linear Model, which do not make any assumptions about the distributions of the scores. Analyses with VSWM and PLTM as ordinal scale dependent measures, and with Age and Hear as independent variables, in the same way as for the data in Figure 1, Table 3, showed exactly the same pattern of significant effects as the ANOVA analyses. For VSWM the effects of Age and Hear were significant with *p*s < 0.000 and the *p*-value of their interaction was 0.025. For PLTM the effects of Age and Hear were also significant with *p*s < 0.000, but the *p*-value of their interaction was >0.10. It was also the case in this SPSS Generalized Linear Model that the effect of Age was about equal for VSWM and PLTM. However, for VSWM the effect of Hear was much weaker than that of Age, while for PLTM the effect of Hear was more substantial than for Age. Thus, in the non-parametric tests we show the same relative effects as those reported from the separate parametric ANOVA analyses as well as from the effect sizes reported. Finally, there is a notable difference in the basic original scales for PLTM and VSWM.

Prospective long-term memory is based on a trichotomization (correct on first attempt, correct at a subsequent attempt, not correct at first or following attempts), while the scale for VSWM was number of errors on and interval scale from 0 to 15. Thus, there was a substantial underestimation of the actual number of errors made in the PLTM task. In spite of this underestimation, poor functional hearing turned out to be significantly related to PLTM, which makes the result even more striking in light of the main hypothesis of the present paper.

## General discussion

The focal finding of this study is that functional hearing loss is clearly related to visuospatial episodic long-term memory (PLTM). This result is important for several reasons.

First, it shows that the negative effect of functional hearing loss is not restricted to mechanisms coupled to auditory perceptual degradation (Schneider et al., 2002, 2010) or to consumption of attention resources due to a compromised auditory signal (Tun et al., 2009; Verhaegen et al., 2014). Although the results in the Rönnberg et al. (2011) study already generalized to verbal tasks with alternative kinds of encoding than the purely auditory or audiovisual (i.e., using motor encoding, Nyberg et al., 1992), the present study has taken a further significant step: here, we demonstrate a robust effect of hearing loss that generalizes to visuo-spatial encoding and subsequent memory retrieval of these kinds of stimuli. Therefore, the negative effects are more pervasive in terms of encoding modality than previously imagined or documented (cf. Rönnberg et al., 2011).

Second, the results replicate the Rönnberg et al. (2011) result showing a stronger impact of hearing loss on episodic long-term memory function rather than on short-term/working memory. The effect size for the poor hearers compared to the normal hearers is substantial (in between medium and large) for PLTM but not for VSWM. Subsequent analyses of subgroups of the poor hearers also showed that the worst subgroup differed from the bad subgroup, but at this level of detail the effect size is relatively low.

Third, the analysis of VSWM revealed a negative effect of functional hearing status, but the relative effects are small and much smaller than for the PLTM task. This finding fits with the overall picture of results from Verhaegen et al. (2014), who also found (significant) negative effects of mild hearing loss on certain short-term memory tasks. Nevertheless, this is also in line with the claim (Rönnberg et al., 2011) that there should be a relatively stronger effect of hearing loss on episodic long-term memory compared to short-term or working memory, mainly because mismatches would reduce the number of times the episodic long-term memory system would be used for encoding, storage and retrieval (Rönnberg et al., 2013).

Fourth, as the effect of using a hearing aid had a relatively positive (error-reducing) effect on the visuospatial working memory task but not on the episodic long-term memory task, the results mimic the Rönnberg et al. (2011) data in that all participants wore hearing aids in that sample—and the negative effect of hearing loss only persisted for semantic and episodic long-term memory. Thus, one more general interpretation of the two sets of results is that there is an effect of hearing loss on short-term memory and long-term memory, the effect is smaller for short-term memory or working memory, and can be at least potentially be compensated for by the use of hearing aids for the poor hearers. This pattern of results agrees with the recent data by Verhaegen et al. (2014) where negative effects of hearing loss were found even in short-term memory tasks, but note that hearing aids were not used by the participants in that study sample.

A counterargument against the positive effect being due to the use of hearing aids as such would be to reverse causality as follows: if good memory were causing people to get and use hearing aids, the group with normal functional hearing who used hearing aids would have better memory. However, since this was not the case (cf. Figure 2) and the poor hearers with hearing aids do have better working memory, then it is likely that the hearing aid is reducing the effect of hearing loss on working memory, and possibly also compensating for the loss as shown by the relative improvement seen for the poor hearers compared to normal hearers.

Understanding the benefit provided by hearing aids (although constrained and small) rests on the fact that functional hearing loss affects PLTM and hearing aid benefit VSWM, i.e., both variables affect the two memory systems selectively. In this study, it happened with a visuospatial VSWM task, but similar results could have been found with an auditory WM task, that is, the general picture that is emerging is that of multimodal processing. The important aspect is the difference in basic cognitive mechanisms underpinning the two tasks, and how other variables latch on to the different properties of those two memory systems.

However, it is also important to note that there could be some initial selection bias relating to individual stages of acceptance of the hearing loss, with the motivation to change and to actively seek help (Manchaiah et al., 2014). Furthermore, yet another interpretation is that the persons who were poor hearers had worn their hearing aids for longer periods of time than the other groups (as hearing loss is usually progressive), and therefore they had developed compensatory skills. However, since the use of a hearing aid did not improve episodic long-term memory, the potential benefit from wearing a hearing aid is relatively *restricted* to VSWM and the effect size was also low. This is also in general agreement with Rönnberg et al. (2011), where we also observed negative effects of hearing loss on episodic long-term memory despite the fact that all participants wore hearing aids. Finally, it is also possible that some hidden cognitive capacity that is not tested in the UK Biobank data set is responsible for the observed interaction. Future research may be more hypothesis-driven in this respect.

Fifth, background variables such as education and gender interact with age for the PLTM task suggesting that the long-term component demonstrates qualitatively different properties compared to working memory. This generally shows that it is important to consider the type of memory system when we are evaluating background variables. It is suggested here that episodic long-term memory is more dependent on crystallized knowledge such as linguistic competence, which is mediated by education (Nyberg et al., 2012) and gender expectations (Lundervold et al., 2014). That kind of competence can also help in decoding the visuospatially presented objects.

Sixth, the negative effect of aging is pervasive across memory systems in the current study, i.e., for both VSWM and PLTM. What we found in Rönnberg et al. (2011) was that hearing loss displayed a negative effect on episodic long-term memory, even when age was statistically controlled for. This is also what we find here: poor hearers are especially prone to error in the PLTM task.

Seventh, the details of the results also show that the relative weighting of the impact of age and hearing loss plays out differently for the two memory tasks. Age is relatively more important for VSWM than for PLTM while hearing loss has a relatively more adverse effect on PLTM than on VSWM. Thus, age and poor hearing play at least partially different roles and may also rely on different mechanisms (Rönnberg et al., 2011).

Eighth, Peelle et al. (2011) have shown that individual differences in hearing acuity (pure tone thresholds) predict activation of bilateral superior temporal regions during auditory sentence comprehension, and that the loss of gray matter is proportional to the degree of audiometric hearing loss, especially in the right auditory cortex. A recent study by Lin et al. (2014) shows that declines in regional brain volumes over 6.4 years are associated with hearing loss, especially in the *right* temporal lobe (superior temporal gyrus, middle temporal gyrus and inferior temporal gyrus), and that this decline is comparable to loss of brain volume in participants with diagnosed mild cognitive impairment (Driscoll et al., 2009). This result is also in line with the previous study by Lin et al. (2011), using a follow-up period that was twice as long, and showing that the risk of developing Alzheimer’s disease is related to hearing loss. However, with our current state of knowledge, it may be too speculative to assume that atrophy in the temporal lobe *also directly* affects visuospatial processing, especially for the PLTM task. Thus, the challenge for future research is to address the many kinds of functional and multimodal brain compensations that may occur due to temporal lobe atrophy, and which also lead to selectivity at the memory systems level.

Ninth, the important aspect here is that we replicate the selectivity *predicted* by the Ease of Language Understanding (ELU) model in the relationship between hearing loss and working memory on the one hand, and episodic long-term memory on the other for different types of tasks (cf. Rönnberg et al., 2011). Again, this effect occurs despite the fact that the underlying scale for PLTM is more conservative (but see more under Section Methodological Issues). This kind of selectivity is not predicted by a common cause account. Also, the association between hearing loss and memory system must be considered to be more central, as our peripheral measures of visual acuity (i.e., wearing eye glasses) did not show any distinctive contribution to memory performance, which is perhaps less surprising than the fact that reported eye problems (which may include more central deficits such as amblyopia) did not show any relationship either. If this line of reasoning is correct, then we may argue for a hearing loss-related central and multimodal mechanism that explains the PLTM decline (Rönnberg et al., 2013) rather than a hypothesis claiming that neural degeneration in general affects both vision and audition in tandem with a general cognitive decline (i.e., the common cause hypothesis, see e.g., Lindenberger and Ghisletta, 2009). However, our claim of a central mechanism should be considered with due caution. One point is that there was no fine-grained or advanced measure of visual acuity/spatial resolution in the UK Biobank database, hence potential associations with visual processing may be underestimated (cf. Humes et al., 2013). Another related point is about causality: even if our hypothesis is about hearing as the independent variable, it is in principle possible that a degradation of visuospatial functions (affecting visuo-spatial memory) may have caused a functional hearing loss. However, the literature on brain tissue degeneration (e.g., Peelle et al., 2011; Lin et al., 2014) suggests that there are right-hemisphere effects that are caused by hearing loss and related to its severity, and again, at least in this study, we do not see any signs of a reversed causality.

Tenth, summarizing across the findings of the current and the Rönnberg et al. (2011) study, functional hearing loss seems to affect episodic long-term memory in general, irrespective of encoding modality, which is why we see effects in visuospatial tasks in the present study, and in Rönnberg et al. (2011) for motor, visual and auditory encoding. The causal nature of the effects needs, however, to be verified in longitudinal studies.

Overall, the large sample in the current study has been helpful in detecting substantial effect sizes related to functional hearing losses. Importantly, it should be noted that these effects apply to non hearing-aid users in the main analyses, suggesting that even relatively mild functional hearing losses do indeed suggest early deterioration of episodic long-term memory function in particular. Altogether, considering the current state of knowledge, including our previous finding that hearing aid wearers show episodic long-term memory deficits related to degree of hearing loss (e.g., Rönnberg et al., 2011), as well as the fact that decline in memory functions represents an important and integral part of dementia and that hearing impairment is related to a substantially increased risk of dementia of Alzheimer type (e.g., Lin et al., 2011), we suggest that the current result is very important from a public health perspective.

### Methodological issues

It could be argued that the DTT is confounded by a* short-term memory* component (as perception *and* recall of digit triplets are required). If the short-term or working memory component was crucial, one would then predict that DTT performance should co-vary with VSWM and not with PLTM. Digit triplet test performance did not co-vary with VSWM. The reason for the lack of an association with VSWM could be that a “load” of a digit triplet is clearly below what is typically given as the normal digit span size (i.e., 7 ± 2). Instead, the DTT variable predicted a decline in PLTM. This kind of double dissociation represents evidence in favor of an interpretation of the present results in terms of a negative effect of functional hearing loss on episodic long-term memory, as outlined by the ELU model (Rönnberg et al., 2011).

It is also clear that there is little reason to believe that the DTT is confounded specifically by semantic *long-term memory* processes (Moore et al., 2014). The DTT has been found to be correlated highly with both an adaptive speech-in-noise test and audiometric testing: the primary interpretation is that it is an auditory speech component that is shared, not a cognitive or linguistic component (cf. Smits et al., 2004). Second, the DTT calls on stored knowledge of a small set of overlearned phonologically dissimilar items with *limited* semantic content whose representation is unlikely to change as a function of either hearing loss or age-related cognitive change. Third, the response format (a touch pad on the screen with the digits laid out) acts as a reminder of the set of available items. Fourth, it is currently unknown how central and peripheral auditory factors play out in the DTT. Further research is needed (cf. Moore et al., 2014), and it would be of interest in the future to investigate the association between hearing and memory using both threshold and functional hearing data.

Another concern that may be raised against the selectivity in the effect of hearing loss on memory systems is the possibility that the results may be confounded by *task difficulty*. However, the PLTM-task was *less* difficult than the VSWM-task in terms of how many percent of the participants produced a correct response on the first trial (80.6 for the PLTM and 7.1% for the VSWM-task). Also, the range of the raw values of number of errors were three for PLTM (0, 1 and 2, or more) and 16 for VSWM (0–15). The logarithmic ranges and means were: VSWM range 0.00–2.77, mean 1.40–0 errors = 7.1%; PLTM range 0.69–1.39, mean −0.78–0 errors = 80.6%. Again, the PLTM task was less difficult than the VSWM task, had fewer steps, and was less sensitive, but still produced significant differences with substantial effect sizes due to functional hearing loss. Reliability estimates are not available from the UK Biobank resource. If we had observed the opposite pattern, viz. that functional hearing loss was associated with larger effects for the VSWM task, then it could have been argued that the effect (at least partially) was due to greater task difficulty that provoked the negative memory effect. In all, it seems unlikely that aspects related to task difficulty could explain the results obtained in the current study.

Finally, visuospatial memory function was *not* related to subjective ratings of hearing disability collected in the UK Biobank database, which suggests that the obtained effects may be based on the loss and objectively determined by an audiogram or by an objective test such as the DTT (Rönnberg et al., 2011; Dawes et al., 2014). Likewise, recent data show that perceived effort in quiet and noise in work-related tasks is hardly ever related to a whole range of cognitive capacities relevant for speech understanding in noise (Hua et al., 2014). This may point to a more general issue regarding ratings of hearing problems and/or effort ratings as predictors of memory or perceptual functions. Several factors may play a role here: it may be the case that the ratings must involve an explicit component of the function under scrutiny and that the function *per se* is explicit (see Rudner et al., 2012; Ng et al., 2013). In the current case, the rating of hearing disability may be too coarse (binary) to measure the explicit functions tapped by VSWM and PLTM. It may also be the case that these types of tasks are less representative of everyday memory problems involved in subjective experiences of hearing problems.

## Conclusion

In all, connecting the memory systems hypothesis with the demands of the visuospatial processing in the memory tasks, the putative negative long-term effect of functional hearing loss is more pronounced for episodic long-term memory (i.e., for PLTM) than for working memory or short-term memory (i.e., for VSWM). This is in line with the ELU prediction about mismatch and relative use/disuse of memory systems (Rönnberg et al., 2011). There may also be a biological basis for a transfer effect from functional hearing loss to episodic long-term memory, including visuospatial and other kinds of multimodal memory encoding formats. It remains for future research to show how e.g., hearing loss-related brain atrophy in the right temporal lobe is associated with general episodic memory deficits.

## Conflict of interest statement

The authors declare that the research was conducted in the absence of any commercial or financial relationships that could be construed as a potential conflict of interest.
